# The diagnostic accuracy of CC chemokine ligand 23 for Kawasaki disease

**DOI:** 10.3389/fimmu.2026.1758367

**Published:** 2026-02-18

**Authors:** Lifen Rao, Jinwen Liao, Xin Guo, Qiuming Miu, Yinbi Zheng, Weiping Xun

**Affiliations:** 1 Department of Pediatric Rehabilitation, Longgang District Maternity & Child Healthcare Hospital of Shenzhen City (Longgang Maternity and Child Institute of Shantou University Medical College), Shenzhen, Guangdong, China; 2 Department of Pediatrics, Longgang District Maternity & Child Healthcare Hospital of Shenzhen City (Longgang Maternity and Child Institute of Shantou University Medical College), Shenzhen, Guangdong, China; 3 Department of Neonatology, Longgang District Maternity & Child Healthcare Hospital of Shenzhen City (Longgang Maternity and Child Institute of Shantou University Medical College), Shenzhen, Guangdong, China; 4 Department of Clinical Laboratory, Longgang District Maternity & Child Healthcare Hospital of Shenzhen City (Longgang Maternity and Child Institute of Shantou University Medical College), Shenzhen, Guangdong, China; 5 Department of Pediatrics, Shenzhen Longhua District Maternity and Child Healthcare Hospital, Shenzhen, Guangdong, China

**Keywords:** biomarkers, CC chemokine ligand 23, diagnostic accuracy, gene expression omnibus database, Kawasaki disease

## Abstract

**Background:**

Kawasaki Disease (KD) is an acute inflammatory disease that primarily affects children. Without timely treatment, it may lead to severe cardiovascular complications. Currently, the lack of specific biomarkers complicates its early diagnosis.

**Methods:**

This study focuses on investigating the diagnostic value of C-C motif chemokine ligand 23 (CCL23) protein in distinguishing KD from other similar diseases through gene expression profiling analysis and biomarker detection. Firstly, methods such as differentially expressed genes (DEGs) analysis, protein-protein interaction (PPI) network construction, and pathway enrichment analysis are employed to identify relevant Hub genes. Subsequently, Western blot technology is used to detect the expression of CCL23 protein in plasma, so as to externally validate the diagnostic value of the aforementioned relevant Hub genes in differentiating Kawasaki disease from other similar diseases.

**Results:**

We identified 11 significant hub genes and found that the concentration of CCL23 in the KD group was significantly higher than that in the febrile control group and the healthy control group. Further receiver operating characteristic (ROC) analysis showed that CCL23 exhibited good sensitivity and specificity in distinguishing Kawasaki Disease from other diseases. Meanwhile, pathway enrichment analysis revealed that CCL23 was upregulated in the cytokine-cytokine receptor interaction pathway, suggesting that it may play an important role in immune-inflammatory responses.

**Conclusions:**

Although this study has limitations such as insufficient sample size and lack of long-term follow-up, the results provide new insights into CCL23 as a potential biomarker for KD. Future studies should further validate these findings and explore their application in clinical practice to improve the early diagnosis rate of KD.

## 1 Introduction

Kawasaki disease (KD) is an acute systemic vasculitis that primarily affects children, and its etiology has not yet been fully elucidated. This disease is more common in children under 5 years of age and is the leading cause of acquired heart disease in children in developed countries. Due to the fact that it may cause coronary artery lesions and even aneurysms during the acute phase, it carries a relatively high risk of disability and mortality (1, 2). The typical clinical manifestations of KD include persistent fever, rash, conjunctivitis, oral mucosal changes, and cervical lymphadenopathy. However, these symptoms can also be observed in a variety of pediatric diseases, which poses significant challenges to clinical diagnosis (3, 4). If timely diagnosis and intervention are not provided during the acute phase, patients are highly likely to develop severe cardiovascular complications, imposing significant psychological and economic burdens on the children and their families (1).

Although the epidemiological characteristics of KD vary by geographical region and ethnicity, its incidence worldwide has been on an annual upward trend, which is particularly prominent in Asia (5). In East Asian countries such as China, Japan, and South Korea, the incidence of KD is much higher than that in Europe and North America, and the age of onset has shown a younger trend in recent years (2). With the outbreak of large-scale infections such as the COVID-19 pandemic, some children have developed multisystem inflammatory syndrome in children (MIS-C), which is highly similar to KD, further increasing the complexity of clinical differential diagnosis (3, 5). Current diagnosis mainly relies on clinical manifestations and routine laboratory tests, but these indicators lack sufficient specificity and are easily confused with bacterial infections, viral infections, autoimmune diseases, and other conditions (3). Therefore, optimizing the early diagnostic process and exploring more specific molecular markers are of great practical significance for improving the prognosis of KD.

Currently, researchers have focused on the molecular mechanisms of KD, such as immune-inflammatory responses, genetic susceptibility, and cytokine networks (2, 6). A large body of evidence indicates that the levels of inflammatory cytokines in children with KD are significantly elevated, and the abnormal expression of related genes is closely associated with the occurrence and progression of the disease (2). In recent years, advances in gene expression profiling technology have provided a powerful tool for uncovering the molecular characteristics of complex diseases. Through high-throughput sequencing of peripheral blood or lesion tissues, it is possible to systematically screen for differentially expressed genes (DEGs) related to KD, laying a foundation for the study of pathogenesis and the screening of biomarkers (7). Protein-protein interaction (PPI) network analysis further reveals the regulatory relationships of key molecules and potential signaling pathways; these methods have been applied in the molecular typing and diagnosis of various inflammatory and autoimmune diseases (7, 8).

However, despite existing studies having revealed KD-related inflammatory responses and several candidate molecules, highly sensitive and specific biomarkers for clinical differential diagnosis remain lacking—particularly evident gaps persist in distinguishing KD from pediatric diseases with similar clinical manifestations, such as bacterial infections, viral infections, juvenile idiopathic arthritis (JIA), and Henoch-Schönlein purpura (HSP) (3, 9). Some studies have attempted to screen candidate molecules based on genomic, transcriptomic, or proteomic data, but most suffer from limitations including small sample sizes, insufficient validation, or lack of multicenter data support. Additionally, how to effectively connect bioinformatics findings with practical clinical detection methods is also a key barrier to the clinical translation of molecular biomarkers (10, 11).

Based on the aforementioned research progress and existing limitations, this study intends to adopt a systems biology perspective and comprehensively apply multi-level methods, including gene expression profiling analysis, PPI network construction, and pathway enrichment analysis, to identify and validate specific molecular biomarkers for KD. Special attention will be paid to the application value of the chemokine C-C motif chemokine ligand 23 (CCL23) in the differential diagnosis of the disease. As an inflammation-related chemokine, changes in the expression and functional effects of CCL23 have been reported in various immune-related diseases; however, its role in KD and its potential as a diagnostic molecular biomarker have not yet been systematically evaluated and confirmed (2, 11).

Specifically, based on public databases and in-house samples, this study will first conduct gene expression profiling analysis of peripheral blood from patients with KD and its common differential diagnosis diseases to screen for DEGs specifically associated with KD. Subsequently, combined with PPI network and biological pathway enrichment analysis, potential core regulatory molecules and key signaling pathways will be identified. Finally, through experimental methods such as protein quantitative detection, candidate biomarkers (e.g., CCL23) will be validated in clinical samples, and their ability to distinguish KD from diseases with similar symptoms will be evaluated. This study aims to provide a new molecular tool for the early and accurate diagnosis of KD, as well as a theoretical basis for revealing its pathogenesis and exploring individualized treatment strategies.

## 2 Methods

### 2.1 Data source and DEGs

After searching for the keywords “Kawasaki disease” and “Homo sapiens” on the website https://www.ncbi.nlm.nih.gov/geo, the series data GSE73464 from the Gene Expression Omnibus (GEO) database was selected for differential gene expression analysis (12, 13). This dataset was detected using the GPL10558 platform and includes a total of 55 healthy control samples, 52 febrile control samples defined as bacterial infections, 94 febrile control samples defined as viral infections, 66 febrile control samples defined as juvenile idiopathic arthritis (JIA), 18 febrile control samples defined as Henoch-Schönlein purpura (HSP), and 77 KD patient samples.

To achieve dataset normalization, we employed the quantile normalization method in the limma (Linear Models for Microarray Data) R package and performed log_2_ transformation (14). Following normalization of the dataset, DEGs were screened according to the criteria of P-value < 0.05 and |log_2_ fold change| > 1. The results of the differential analysis were visualized using the ggplot2 [3.4.4] package.

### 2.2 PPI network and pathway enrichment analysis

We used the STRING database to construct and analyze the PPI network of the selected modules. R (version 4.2.1) with the clusterProfiler [4.4.4] and GOplot [1.0.2] packages was employed for gene pathway enrichment analysis, and the most critical signaling pathways were screened based on the Kyoto Encyclopedia of Genes and Genomes (KEGG) database. After performing ID conversion on the input molecular list, the clusterProfiler package was used to conduct enrichment analysis, and the GOplot package was applied to calculate the z-score corresponding to each enriched term using the provided molecular values.

### 2.3 Hub genes for distinguishing KD from diseases with similar symptoms

Using the GSE73464 expression microarray dataset, we analyzed the gene differences between KD and a group of diseases with similar symptoms, including bacterial infections, viral infections, JIA, and HSP. The intersection of differentially expressed genes between the KD group and other disease groups, as well as between the KD group and the healthy control group (defined as: |log_2_ fold change| > 1 and P-value < 0.05 in the KD group relative to all control groups) was regarded as potential hub genes.

### 2.4 Research subjects and study samples in validation experiments

Blood samples were collected from children diagnosed with KD, children with fever due to other causes, and age- and gender-matched healthy children who visited the Maternal and Child Health Hospital of Longgang District, Shenzhen. These samples were placed in blood collection tubes containing ethylenediaminetetraacetic acid (EDTA). After the blood samples were centrifuged (at 1800 rpm for 10 minutes), the plasma was extracted and stored in a -80 °C refrigerator. Meanwhile, the quantitative detection of CCL23 protein was conducted using the Human CCL23 ELISA Kit (Boster Biological Technology, Product No.: EK1224) in strict accordance with the kit instructions. This study strictly adhered to the ethical guidelines approved by the Medical Ethics Committee of the Maternal and Child Health Hospital of Longgang District, Shenzhen, as well as the Declaration of Helsinki. Written informed consent was obtained from the guardians of all participants prior to sample collection. The study participants included 23 children with Kawasaki Disease, 16 children with fever due to other causes, and 16 healthy children, all of whom were under 5 years of age. In addition, we also collected the demographic information, clinical data, laboratory test results and echocardiographic findings of KD patients, as well as the disease types and pathogen detection results of children in the febrile control group (details are provided in **Supplementary Tables S1**-**S3**). Consistent with previous studies, we validated our findings using other KD-related GEO datasets GSE18606 and GSE68004, and the results are presented in **Supplementary Figure S1**.

### 2.5 Relevant definitions in validation experiments

The Kawasaki Disease Group (KD Group) refers to children who have had a persistent fever for at least 5 days, accompanied by at least 4 out of the following 5 major clinical features (15):

1. Rash;
2. Bilateral non-exudative conjunctivitis;
3. Oral mucosal changes (e.g., cheilitis with fissuring (dry and cracked lips), strawberry tongue);
4. Extremity changes (e.g., erythema and edema of the hands and feet during the acute phase, desquamation of the skin on the fingertips and toe tips during the convalescent phase);
5. Non-suppurative cervical lymphadenopathy (usually with a diameter greater than 1.5 centimeters).

The fever control group (FC Group) comprised children who were admitted to the hospital during the same period as the Kawasaki Disease patients, matched for age and gender, and diagnosed with fever caused by non-Kawasaki Disease etiologies.

The healthy control group (HC Group) included children who underwent health check-ups at the Child Health Care Outpatient Department during the same period, were confirmed to be healthy, and were also matched for age and gender.

### 2.6 Statistical analysis

Statistical analysis and data visualization were performed using R (version 4.2.1) and STATA 18.5 MP (StataCorp LLC). We presented the baseline characteristics of all participants grouped into the KD, FC, and HC groups. For quantitative data, values were described using mean ± standard deviation (SD), or median (P25–P75), and the statistical significance of differences between groups was assessed by the student’s t-test, one-way analysis of variance (ANOVA), or Kruskal–Wallis rank sum test. For qualitative data, values were expressed as frequency (percentage), and the statistical significance of differences between groups was evaluated with the chi-square test.

The Mann-Whitney U test was also used to compare the concentrations of CCL23 protein among the KD, FC, and HC groups. The diagnostic efficacy of CCL23 in distinguishing between the KD group and FC group, KD group and HC group, as well as the KD group and the combined FC and HC groups was evaluated by plotting receiver operating characteristic (ROC) curves and calculating the area under the curve (AUC). Additionally, the 95% Confidence Intervals (CI) for AUC were calculated. The Youden’s Index was calculated to determine the optimal diagnostic cutoff value: sensitivity, specificity, positive predictive value (PPV), and negative predictive value (NPV) were computed accordingly. ROC analysis of the data was conducted using pROC [1.18.0] in R, and the results were visualized with ggplot2 [3.4.4] in R. The criterion for statistical significance was set at a P-value < 0.05.

## 3 Results

### 3.1 Identification of DEGs and hub genes

**Figure 1:**
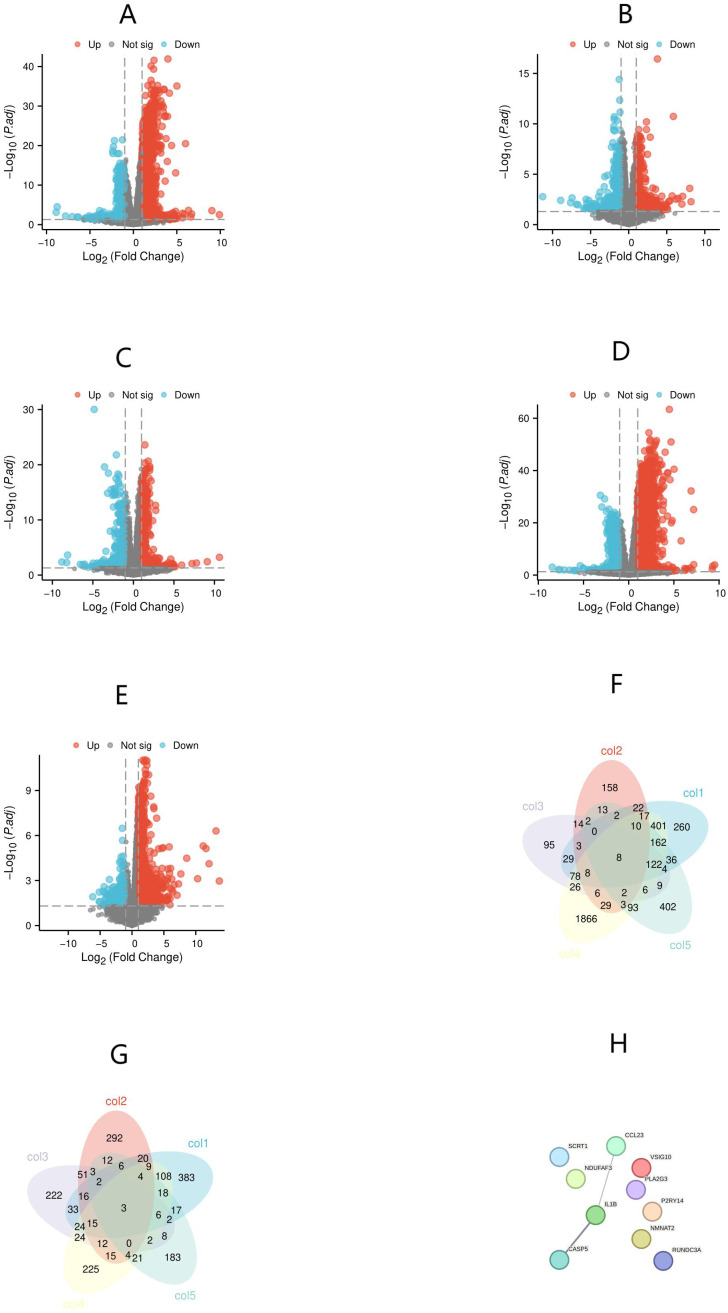
Identification of differentially expressed genes (DEGs) and hub genes, and protein-protein interaction (PPI) network analysis of hub genes. **(A)** Kawasaki Disease (KD) versus healthy controls; **(B)** KD versus bacterial infections; **(C)** KD versus viral infections; **(D)** KD versus juvenile idiopathic arthritis (JIA); **(E)** KD versus Henoch-Schönlein purpura (HSP). **(F)** Hub genes with differentially upregulated expression; **(G)** Hub genes with differentially downregulated expression; **(H)** PPI network analysis of hub genes.

To compare the gene expression profiles of KD samples with those of febrile controls and healthy controls, we screened all DEGs using thresholds of absolute log2 Fold Change (|log2FC|) > 1 and p-value < 0.05. These DEGs were then presented in volcano plots (**Figures 1A-E**). Through overlapping analysis, we identified eight upregulated and three downregulated common hub genes (**Figures 1F, G**). PPI network analysis of these 11 hub genes revealed interactions among three genes: CASP5, IL1B, and CCL23 (**Figure 1H**). Additionally, CASP5, IL1B, and CCL23 are all hub genes with significantly upregulated expression. Interestingly, the diagnostic value of IL1B and CASP5 for KD has been reported in existing literature (14, 16). Therefore, the next step of this study will focus on verifying the value of CCL23 in distinguishing KD from febrile controls and healthy controls.

### 3.2 Enrichment analysis of DEGs

**Figure 2:**
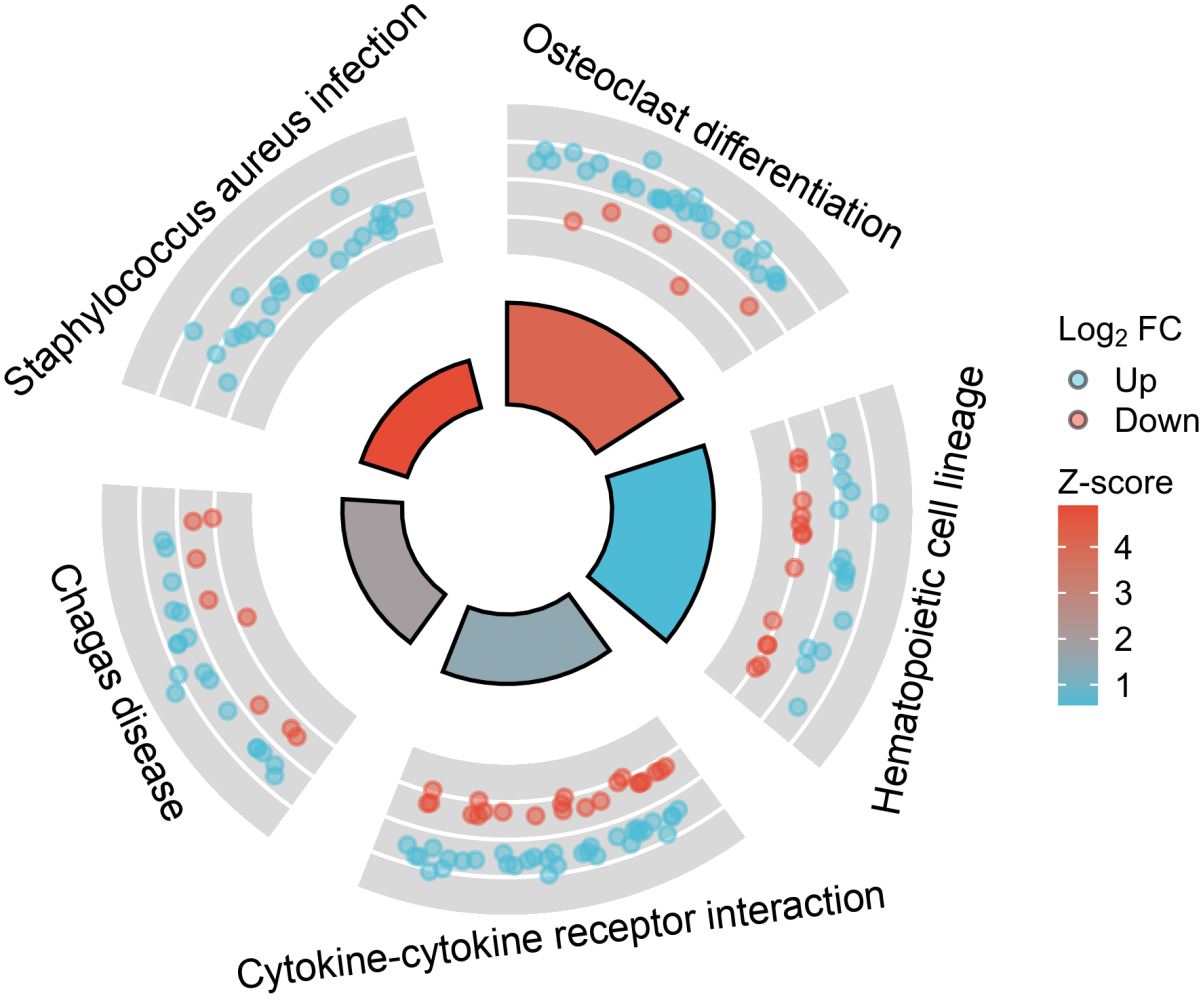
The top five most significantly enriched Kyoto Encyclopedia of Genes and Genomes (KEGG) pathways among the differentially expressed genes (DEGs) between Kawasaki Disease (KD) and healthy controls.

To understand the functional enrichment of DEGs, we analyzed KEGG pathway enrichment of DEGs between KD samples and febrile control samples, as well as between KD samples and healthy control samples. Among the DEGs between KD and healthy controls, 22 significantly enriched KEGG pathways were identified with an adjusted p-value below 0.05, and the top five most significantly enriched pathways are presented in **Figure 2**. The differentially upregulated CCL23 exactly belongs to the KEGG entry “Cytokine-cytokine receptor interaction”. In contrast, no significantly enriched KEGG pathways were found in the DEGs between KD and bacterial infection, viral infection, JIA, or HSP, as all had adjusted p-values equal to or greater than 0.05.

### 3.3 Participant inclusions in validation experiments

**Table 1** presents the distribution of demographic and clinical characteristics, as well as CCL23 concentrations, among the three groups of participants. Statistically significant differences were observed among the KD, FC, and HC groups in peripheral blood white blood cell count, hemoglobin level, neutrophil count, the ratio of neutrophil to lymphocyte counts, and plasma CCL23 concentration (P < 0.05). Additionally, plasma high-sensitivity C-reactive protein and serum albumin levels differed significantly between the KD and FC groups (P < 0.05). No statistically significant differences were found among the KD, FC, and HC groups in sex, age, peripheral blood platelet count, lymphocyte count, or the ratio of platelet to lymphocyte counts (P ≥ 0.05). Sixteen febrile control children were mainly affected by respiratory diseases, with Mycoplasma and respiratory viruses as the common pathogens, and nearly half had no detectable pathogens (**Supplementary Table S1**). All 23 KD patients were diagnosed with complete KD with 8.7% (2/23) having coronary artery involvement and no cases of Intravenous Immunoglobulin (IVIG) resistance (**Supplementary Table S2**, **S3**).

**Table 1:** Baseline clinical characteristics and CCL23 concentrations of participants in the three groups.

Characteristics	KD (n=23)	FC (n=16)	HC (n=16)	P-value
Male sex, n (%)	11 (47.83)	11 (68.75)	6 (37.50)	0.194
Age (months), mean ± SD	17.52 ± 11.34	21.38 ± 10.72	21.88 ± 12.98	0.440
Peripheral blood white blood cell count (×10^9^/L), mean ± SD	13.88 ± 3.69	9.60 ± 4.39	8.80 ± 2.97	<0.001
Peripheral blood hemoglobin content (g/L), mean ± SD	108 ± 8.20	119.81 ± 10.39	122.81 ± 9.12	<0.001
Peripheral blood platelet count (×10^9^/L), mean ± SD	373.09 ± 106.13	303.88 ± 138.90	321.13 ± 76.01	0.127
Peripheral blood lymphocyte count (×10^9^/L), mean ± SD	3.68 ± 1.82	3.57 ± 1.64	4.36 ± 1.78	0.385
Peripheral blood neutrophil count (×10^9^/L), mean ± SD	8.94 ± 3.39	5.18 ± 3.26	3.63 ± 2.26	<0.001
Ratio of peripheral blood neutrophil count to lymphocyte count, median (P25–P75)	2.56 (1.42-3.58)	1.54 (0.99-2.19)	0.65 (0.49-1.50)	<0.001
Ratio of peripheral blood platelet count to lymphocyte count, median (P25–P75)	113.80 (74.85-122.88)	96.19 (61.96-125.32)	74.92 (63.06-108.35)	0.094
Plasma high-sensitivity C-reactive protein content (mg/L), mean ± SD	89.58 ± 46.41	21.96 ± 32.23		<0.001
Serum albumin content (g/L), mean ± SD	37 ± 3.88	42.56 ± 3.35		<0.001
Plasma CCL23 concentration (pg/ml), median (P25–P75)	1702.60 (1176.79-3563.70)	512.38 (361.46-838.66)	191.37 (147.10-234.36)	<0.001

CCL23, CC Chemokine Ligand 23; KD, Kawasaki disease; FC, fever control; HC, healthy control.

### 3.4 Diagnostic accuracy of CCL23 in validation experiments

**Figure 3:**
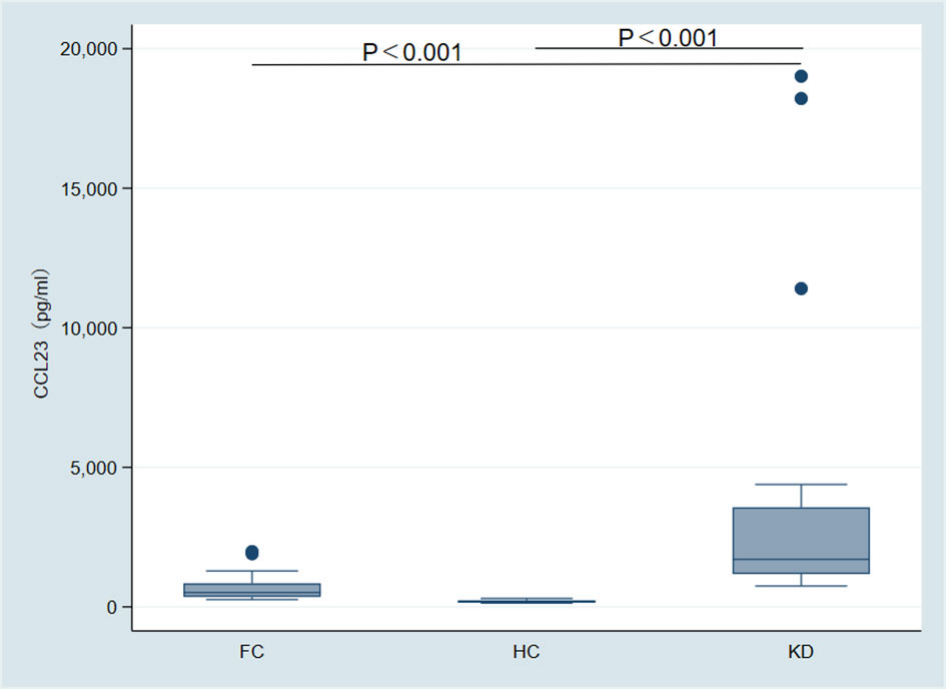
Intergroup comparison of CCL23 protein expression among the Kawasaki disease (KD) group, fever control (FC) group, and healthy control (HC) group.

**Figure 4:**
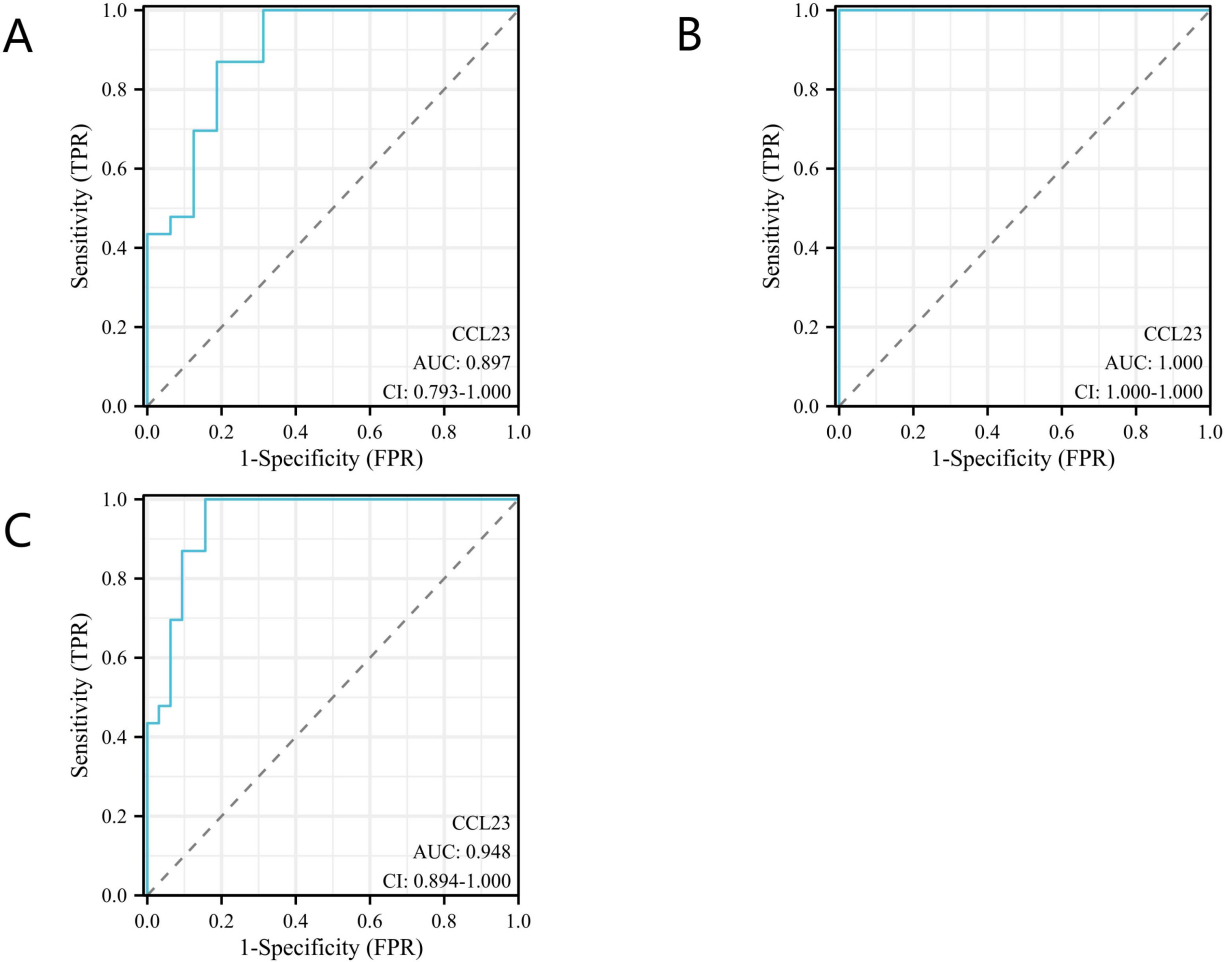
Diagnostic accuracy analysis of CC Chemokine Ligand 23 (CCL23) for Kawasaki disease (KD). **(A)** Comparison between the KD group and the fever control (FC) group; **(B)** Comparison between the KD group and the healthy control (HC) group; **(C)** Comparison between the KD group and the combined FC and HC groups.

The concentration of CCL23 protein in the KD group (1702.60 pg/ml, range 1176.79–3563.70) was significantly higher than in the FC group (512.38 pg/ml, range 361.46–838.66) and the HC group (191.37 pg/ml, range 147.10–234.36). This difference was statistically significant (p < 0.001) (**Figure 3**). ROC analysis showed that the AUC for distinguishing the KD group from the FC group was 0.90 (95% CI 0.79–1.00) (**Figure 4A**). When the diagnostic cut-off value of CCL23 protein concentration was set at 720.17 pg/ml, Youden’s index reached its maximum observed value, resulting in a diagnostic sensitivity of 100%, specificity of 69%, PPV of 82%, and NPV of 100% (**Table 2**). The AUC for distinguishing the KD group from the HC group was 1.00 (95% CI 1.00–1.00) (**Figure 4B**). When the diagnostic cut-off value was set at 524.56 pg/ml, Youden’s index reached its maximum observed value, with a diagnostic sensitivity, specificity, PPV, and NPV all at 100% (**Table 2**). The AUC for distinguishing the KD group from the combined FC and HC group was 0.95 (95% CI 0.89–1.00) (**Figure 4C**). When the diagnostic cut-off value was set at 720.17 pg/ml, Youden’s index again reached its maximum observed value, yielding a diagnostic sensitivity of 100%, specificity of 84%, PPV of 82%, and NPV of 100% (**Table 2**). In the external validation of GEO dataset GSE18606 (n=29), CCL23 expression was significantly elevated in KD patients, showing moderate diagnostic efficacy for KD and enabling distinction between healthy controls and KD cases. In the external validation of GEO dataset GSE68004 (n=162), CCL23 expression was also significantly higher in KD patients compared to healthy controls, but showed no significant difference when compared to infectious diseases, indicating weak diagnostic efficacy for KD.

**Table 2:** Diagnostic accuracy indicators of CCL23 for KD.

Comparisons	AUC (95%CI)	Cut-off values (pg/ml)	Youden’s index	Sensitivity	Specificity	PPV	NPV
KD versus FC	0.90 (0.79-1.00)	720.17	0.69	1.00	0.69	0.82	1.00
KD versus HC	1.00 (1.00-1.00)	524.56	1.00	1.00	1.00	1.00	1.00
KD versus FC+HC	0.95 (0.89-1.00)	720.17	0.84	1.00	0.84	0.82	1.00

CCL23, CC Chemokine Ligand 23; KD, Kawasaki disease; AUC, area under the curve; CI, Confidence Intervals; PPV, positive predictive value; NPV, negative predictive value; FC, fever control; HC, healthy control.

## 4 Discussion

This study aims to explore the diagnostic value of the CCL23 protein in Kawasaki Disease (KD) and its differentiation from other diseases with similar symptoms, such as bacterial infections and viral infections. Through comprehensive gene expression profile analysis and biomarker detection, we identified the unique gene expression characteristics of KD and confirmed the potential of CCL23 in differential diagnosis. The results showed that the expression of CCL23 was significantly upregulated in KD patients, and this finding provides a new perspective for the early clinical diagnosis of KD.

In this study, differential gene expression analysis revealed a set of specifically upregulated and downregulated hub genes in patients with KD. This finding provides a new entry point for understanding its molecular mechanisms. Existing studies have indicated that the pathogenesis of KD is closely associated with the abnormal activation of the immune system. Particularly, changes in the expression of specific genes during the secretion of inflammatory factors and the recruitment of immune cells can drive disease progression (1, 2). The upregulated genes identified in this study show enhanced expression in immune-related pathways, which is highly consistent with the previously reported upregulation of inflammatory factors (such as IL-1, IL-6, etc.) in the acute phase of KD (17, 18). However, some downregulated genes exhibit functional defects related to the maintenance of normal immune homeostasis, which is also reflected in previous literature reports on T-cell and regulatory immune imbalances in KD (19). Compared with the control groups of bacterial infections, viral infections and other immune diseases, the expression profiles of these hub genes show more significant differentiation in this study, suggesting that they are involved in the KD-specific inflammatory signal regulatory network (3). Notably, although previous studies have reported the upregulation of some inflammation-related genes in KD, this study systematically screened and verified multiple novel hub molecular nodes through comprehensive genomic analysis, providing a more targeted molecular basis for subsequent mechanism research and targeted intervention strategies.

PPI network analysis further revealed the synergistic effects among hub genes, particularly the tight interaction between CASP5, IL1B, and CCL23, suggesting their key roles in amplifying inflammatory responses and cytokine cascades. Previous studies have confirmed that IL1B, as a core mediator of inflammatory cascades, can induce vascular inflammation and immune cell infiltration by promoting the expression of downstream cytokines (2, 17). CASP5, on the other hand, plays an important regulatory role in programmed cell death and inflammasome activation, and its upregulation may exacerbate local tissue damage and immune disorders (17). As previously mentioned, the effects and diagnostic value of IL1B and CASP5 in KD have been reported in existing literature (14, 16). As a chemokine, CCL23 has been found to be associated with the recruitment of immune cells (such as monocytes and eosinophils) in various inflammatory and vascular diseases (20, 21). The PPI network in this study showed that these three molecules form a highly interactive network structure in KD, highlighting the mechanism by which multiple factors synergistically drive the inflammatory cascade reaction.

The results of pathway enrichment analysis showed that the cytokine-receptor interaction pathway was significantly enriched in patients with KD. In particular, the upregulation of CCL23 indicates its core role in inflammatory signal transduction. A large number of studies have shown that the levels of various cytokines in the plasma of patients with KD during the acute phase are significantly increased, which are involved in a series of downstream events such as immune cell chemotaxis, activation, and endothelial damage (1, 2). As a chemokine, CCL23 can not only mediate the aggregation of immune cells to inflammatory sites, but also regulate cytokine release and vascular permeability through binding to its receptor CCR1 (20, 22). Similar to the mechanism of CCL23 in other autoimmune and vasculitic diseases, this study found that the upregulation of CCL23 in KD is closely related to the active state of immune inflammatory response (23). In addition, pathway enrichment analysis also verified the involvement of classical inflammatory pathways such as the IL-1 family and TNF, further confirming the molecular mechanism of KD pathogenesis characterized by the synergistic amplification of multiple inflammatory signals. Therefore, the abnormal activation of CCL23 and its related pathways may become a new direction for specific molecular markers and intervention targets of KD.

Regarding the clinical diagnostic value of CCL23, this study showed that its concentration in the peripheral blood of KD patients was significantly higher than that in the febrile control group and the healthy children group. Previous studies have reported that CCL23 is elevated in various inflammatory vascular lesions, acute stroke, and systemic autoimmune diseases, and exhibits high diagnostic sensitivity and specificity (20, 21, 24). A study from Italy demonstrated that CCL23 is significantly elevated in KD patients and markedly decreased following IVIG treatment (25). Studies have found that compared with other similar diseases, the expression of CCL23 in the plasma of children with KD is significantly upregulated (26, 27). This indicates that the elevation of CCL23 may be associated with the pathogenesis of KD across different ethnic backgrounds. A study conducted in Xuzhou, China identified via Olink proteomics technology that CCL23 (AUC: 0.867) exhibits high diagnostic value in distinguishing KD from other febrile diseases, and we verified and supported this finding by combining GEO database analysis with ELISA (26). However, this study did not validate the diagnostic performance of CCL23 in subsequent cytokine detection assays, and its discussion section focused on analyzing the diagnostic value of IL-17 in KD without an in-depth exploration of the diagnostic significance of CCL23. Our external validation results from two U.S.-originated GEO datasets revealed that CCL23 expression levels were significantly elevated in the KD group compared with the healthy group, with moderate diagnostic performance in discriminating between these two groups. Nevertheless, no significant differences in CCL23 expression were observed in KD patients relative to the specific infectious disease group, accompanied by poor diagnostic performance for this differentiation. This indicates that future studies enrolling larger sample sizes, covering more extensive regions and adopting more diversified grouping strategies are still required to validate these findings (28, 29). Consistent with the conclusions of previous studies that CCL23 can serve as a serum biomarker for distinguishing different types of vasculitis and assessing disease activity, this study not only systematically validated this biomarker but also conducted an in-depth investigation into its application advantages in differentiating Kawasaki disease (KD) from diseases with similar clinical manifestations for the first time (22, 23). It is worth emphasizing that existing literature mostly focuses on the diagnostic value of CCL23 in adult systemic inflammation and tumors, while this study applies it to pediatric inflammatory diseases and the differentiation of their subtypes, expanding the boundaries of its clinical translational application. In conclusion, as a downstream effector molecule of inflammatory responses, the plasma expression level of CCL23 not only reflects pathological progression but also provides a new molecular tool for the early and accurate diagnosis of KD.

The main limitation of this study lies in the relatively small sample size, which may restrict the statistical significance of the results and their external generalizability. An insufficient sample size might lead to the failure of effective identification of certain potential biomarkers, thereby affecting our assessment of the true diagnostic capability of CCL23. In addition, the lack of long-term follow-up data prevents us from fully understanding the changes in the disease course of patients with KD and the relationship between CCL23 levels and disease progression, which to some extent weakens the clinical relevance of the study.

## 5 Conclusion

In summary, this study reveals the importance of CCL23 as a potential biomarker for KD, and emphasizes its sensitivity and specificity in the differential diagnosis of KD from other diseases with similar symptoms. Future studies should expand the sample size and integrate long-term follow-up data to verify the reliability of CCL23 in early diagnosis. Meanwhile, specific strategies for its clinical application should be explored to facilitate the timely identification and effective management of KD.

## Data Availability

The original contributions presented in the study are included in the article/Supplementary Material. Further inquiries can be directed to the corresponding author.

## Supplementary material

Supplementary Figure 1 External validation results based on GEO datasets GSE18606 and GSE68004. HC, healthy control; KD, Kawasaki disease; AUC, area under the curve; CCL23, CC chemokine ligand 23. A: comparison between the two groups in the GSE18606 dataset; B: Comparison between the three groups in the GSE68004 dataset; C: ROC curve for distinguishing KD and HC in the GSE18606 dataset; D: ROC curve for distinguishing KD and HC in the GSE68004 dataset; E: ROC curve for distinguishing KD and infectious diseases in the GSE68004 dataset. Infectious diseases include adenovirus infection and Group A streptococcal disease.

Supplementary Table 1 Disease types detected in the group of febrile children.

Supplementary Table 2 Clinical features of KD enrolled patients.

Supplementary Table 3 Echocardiographic values of the coronary arteries expressed in mm and as Z-score of KD patients with coronary involvement.

## References

[1] Zeinali M Frishman WH Aronow WS . Kawasaki disease. Cardiol Rev. (2025). doi: 10.1097/crd.0000000000000959, PMID: 40396727

[2] Bordea MA Costache C Grama A Florian AI Lupan I Samasca G . Cytokine cascade in Kawasaki disease versus Kawasaki-like syndrome. Physiol Res. (2022) 71:17–27. doi: 10.33549/physiolres.934672, PMID: 35043641 PMC8997683

[3] Saez-de-Ocariz M Gámez-González LB Rivas-Larrauri F Castaño-Jaramillo LM Toledo-Salinas C Garrido-García LM . Kawasaki disease mimickers. Pediatr Int. (2021) 63:880–8. doi: 10.1111/ped.14561, PMID: 33249696

[4] Turk S Aydin D Dogan E Levent E Kutukculer N . Periodic fever syndromes: a patient diagnosed with recurrent Kawasaki disease. Cardiol Young. (2020) 30:1009–11. doi: 10.1017/s1047951120001444, PMID: 32524933

[5] Bitsadze VO Grigoreva K Khizroeva JK Pervunina TM Tsibizova VI Tretyakova MV . Novel coronavirus infection and Kawasaki disease. J Matern Fetal Neonatal Med. (2022) 35:3044–8. doi: 10.1080/14767058.2020.1800633, PMID: 32731783

[6] Huang PY Huang YH Guo MM Chang LS Kuo HC . Kawasaki disease and allergic diseases. Front Pediatr. (2020) 8:614386. doi: 10.3389/fped.2020.614386, PMID: 33490002 PMC7817814

[7] Majeed A Mukhtar S . Protein-protein interaction network exploration using cytoscape. Methods Mol Biol. (2023) 2690:419–27. doi: 10.1007/978-1-0716-3327-4_32, PMID: 37450163

[8] Hollander M Do T Will T Helms V . Detecting rewiring events in protein-protein interaction networks based on transcriptomic data. Front Bioinform. (2021) 1:724297. doi: 10.3389/fbinf.2021.724297, PMID: 36303788 PMC9581068

[9] Liu X Zhou K Hua Y Wu M Liu L Shao S . Grisel’s syndrome in Kawasaki disease. Orphanet J Rare Dis. (2020) 15:246. doi: 10.1186/s13023-020-01535-0, PMID: 32917253 PMC7488729

[10] Han Q Pang J Li Y Sun B Ibarlucea B Liu X . Graphene biodevices for early disease diagnosis based on biomarker detection. ACS Sens. (2021) 6:3841–81. doi: 10.1021/acssensors.1c01172, PMID: 34696585

[11] Le Goff C Ladang A Gothot A Cavalier E . Biological markers of inflammation: an update. Rev Med Liege. (2022) 77:258–64., PMID: 35657180

[12] Kuiper R Wright VJ Habgood-Coote D Shimizu C Huigh D Tremoulet AH . Bridging a diagnostic Kawasaki disease classifier from a microarray platform to a qRT-PCR assay. Pediatr Res. (2023) 93:559–69. doi: 10.1038/s41390-022-02148-y, PMID: 35732822 PMC9988687

[13] Wright VJ Herberg JA Kaforou M Shimizu C Eleftherohorinou H Shailes H . Diagnosis of kawasaki disease using a minimal whole-blood gene expression signature. JAMA Pediatr. (2018) 172:e182293. doi: 10.1001/jamapediatrics.2018.2293, PMID: 30083721 PMC6233768

[14] Rahmati Y Mollanoori H Najafi S Esmaeili S Alivand MR . CASP5 and CR1 as potential biomarkers for Kawasaki disease: an Integrated Bioinformatics-Experimental Study. BMC Pediatr. (2021) 21:566. doi: 10.1186/s12887-021-03003-5, PMID: 34895171 PMC8665509

[15] McCrindle BW Rowley AH Newburger JW Burns JC Bolger AF Gewitz M . Diagnosis, treatment, and long-term management of kawasaki disease: A scientific statement for health professionals from the american heart association. Circulation. (2017) 135:e927–e99. doi: 10.1161/cir.0000000000000484, PMID: 28356445

[16] Wang Z Wang Q Jin J Rong X Wu T Qiu H . The diagnostic role of AIM2 in Kawasaki disease. Clin Exp Med. (2021) 21:41–7. doi: 10.1007/s10238-020-00669-6, PMID: 33079289

[17] Shahi A Afzali S Firoozi Z Mohaghegh P Moravej A Hosseinipour A . Potential roles of NLRP3 inflammasome in the pathogenesis of Kawasaki disease. J Cell Physiol. (2023) 238:513–32. doi: 10.1002/jcp.30948, PMID: 36649375

[18] Goel AR Yalcindag A . An update on kawasaki disease. Curr Rheumatol Rep. (2024) 27:4. doi: 10.1007/s11926-024-01167-4, PMID: 39625646

[19] Kaneko K Akagawa S Akagawa Y Kimata T Tsuji S . Our evolving understanding of kawasaki disease pathogenesis: role of the gut microbiota. Front Immunol. (2020) 11:1616. doi: 10.3389/fimmu.2020.01616, PMID: 32793240 PMC7393004

[20] Hao W Liu Q Li X Xu Y Guan W Zhang L . CCL23 is a potential biomarker for antineutrophil cytoplasmic antibody-associated vasculitis. Arthritis Res Ther. (2025) 27:83. doi: 10.1186/s13075-025-03552-5, PMID: 40211307 PMC11983769

[21] Wang X Yang Y Zhao Z Li P Ma C Zhu B . Diagnostic value of serum MIF and CCL23 in the patients with acute cerebral infarction. Clin Lab. (2020) 66. doi: 10.7754/Clin.Lab.2020.200239, PMID: 33180438

[22] Söderlund S Boey D van Midden W Kjellander M Ax K Qian H . Proteomic and transcriptomic screening demonstrates increased mast cell-derived CCL23 in systemic mastocytosis. J Allergy Clin Immunol. (2023) 152:205–13. doi: 10.1016/j.jaci.2023.01.033, PMID: 36813186

[23] Lin H Shen J Zhu Y Zhou L Zhang S Liu Z . Serum CCL23 emerges as a biomarker for poor prognosis in patients with intracerebral hemorrhage. Clin Chim Acta. (2022) 537:188–93. doi: 10.1016/j.cca.2022.10.012, PMID: 36309070

[24] Roderburg C Labuhn S Bednarsch J Lang SA Schneider AT Hammerich L . Elevated serum levels of CCL23 are associated with poor outcome after resection of biliary tract cancer. Mediators Inflamm. (2022) 2022:6195004. doi: 10.1155/2022/6195004, PMID: 36505756 PMC9731746

[25] Cotugno N Olivieri G Pascucci GR Amodio D Morrocchi E Pighi C . Multi-modal immune dynamics of pre-COVID-19 Kawasaki Disease following intravenous immunoglobulin. Clin Immunol. (2024) 267:110349. doi: 10.1016/j.clim.2024.110349, PMID: 39186994

[26] Tu X Chen X Xu L Yang C Li J Liu Y . Serum olink targeted proteomics identifies IL-17A as a prospective inflammatory marker for the prediction and diagnosis of kawasaki disease. J Inflammation Res. (2025) 18:3093–103. doi: 10.2147/jir.S506154, PMID: 40059947 PMC11889999

[27] Ko TM Kuo HC Chang JS Chen SP Liu YM Chen HW . CXCL10/IP-10 is a biomarker and mediator for Kawasaki disease. Circ Res. (2015) 116:876–83. doi: 10.1161/circresaha.116.305834, PMID: 25605650

[28] Fury W Tremoulet AH Watson VE Best BM Shimizu C Hamilton J . Transcript abundance patterns in Kawasaki disease patients with intravenous immunoglobulin resistance. Hum Immunol. (2010) 71:865–73. doi: 10.1016/j.humimm.2010.06.008, PMID: 20600450 PMC2929310

[29] Jaggi P Mejias A Xu Z Yin H Moore-Clingenpeel M Smith B . Whole blood transcriptional profiles as a prognostic tool in complete and incomplete Kawasaki Disease. PloS One. (2018) 13:e0197858. doi: 10.1371/journal.pone.0197858, PMID: 29813106 PMC5973615

