# Supplementary material for: The diagnostic accuracy of CC chemokine ligand 23 for Kawasaki disease
Source: Front Immunol. 2026 Feb 18;17:1758367. doi: 10.3389/fimmu.2026.1758367 (PMC12957167; doi:10.3389/fimmu.2026.1758367)
Supplement: Supplementary Figure 1 — External validation results based on GEO datasets GSE18606 and GSE68004. HC, healthy control; KD, Kawasaki disease; AUC, area under the curve; CCL23, CC chemokine ligand 23. A: comparison between the two groups in the GSE18606 dataset; B: Comparison between the three groups in the GSE68004 dataset; C: ROC curve for distinguishing KD and HC in the GSE18606 dataset; D: ROC curve for distinguishing KD and HC in the GSE68004 dataset; E: ROC curve for distinguishing KD and infectious diseases in the GSE68004 dataset. Infectious diseases include adenovirus infection and Group A streptococcal disease. [file DataSheet1.docx]

Supplementary Material

**Table S1.** Disease types detected in the group of febrile children.

| **Febrile Patients (n=16)** | **Disease types** | **Pathogens** |
| --- | --- | --- |
| FC1 | Bronchopneumonia | Human respiratory syncytial virus |
| FC2 | Bronchitis | Human respiratory adenovirus |
| FC3 | Severe pneumonia | Mycoplasma pneumoniae |
| FC4 | Bronchopneumonia | Negative |
| FC5 | Bronchopneumonia | Negative |
| FC6 | Febrile seizure | Negative |
| FC7 | Bronchopneumonia | Mycoplasma pneumoniae |
| FC8 | Acute tonsillitis | Negative |
| FC9 | Herpangina | Negative |
| FC10 | Bronchitis | Mycoplasma pneumoniae |
| FC11 | Acute pharyngitis | Negative |
| FC12 | Bronchopneumonia | Negative |
| FC13 | Bronchopneumonia | Rhinovirus, Mycoplasma |
| FC14 | Acute suppurative tonsillitis | Negative |
| FC15 | Severe pneumonia | Haemophilus haemolyticus, Human parainfluenza virus |
| FC16 | Acute pharyngitis | Negative |

**Table S2.** Clinical features of KD enrolled patients.

| **Clinical features** | **n (%)** |
| --- | --- |
| Fever | 23 (100%) |
| Conjunctival injection | 23 (100%) |
| Rash | 21 (91.3%) |
| Mucosal changes | 22(95.6%) |
| Extremity changes | 16 (69.6%) |
| Cervical lymphadenopathy | 20 (87.0%) |
| Coronary artery involvement | 2(8.7%) |
| **Clinical presentation** | **n (%)** |
| Complete KD/Incomplete KD | 23/0 |
| Time to diagnosis, median days (range) | 5.0 (1.0-9.0) |
| **Treatment** | **n (%)** |
| Corticosteroids | 1 (4.35%) |
| Corticosteroids bolus | 7 (16.3%) |
| ASA | 43 (100%) |
| IVIG | 43 (100%) |
| IVIG resistance | 0 (0%) |

Table S3. Echocardiographic values of the coronary arteries expressed in mm and as Z-score of KD patients with coronary involvement.

| ID patient | **LM mm** | **LM**  **Z-score** | **LAD**  **mm** | **LAD**  **Z-score** | **RCA**  **mm** | **RCA**  **Z-score** |
| --- | --- | --- | --- | --- | --- | --- |
| KD01 | 17 | 1.719 | N/A | N/A | 17 | 0.663 |
| KD02 | 19 | 1.500 | N/A | N/A | 17 | -0.963 |
| KD03 | 29 | 3.120 | 30 | 5.046 | 37 | 6.287 |
| KD04 | 22 | 0.800 | N/A | N/A | 22 | 1.634 |
| KD05 | 26 | 1.221 | N/A | N/A | 15 | -1.139 |
| KD06 | 22 | 0.655 | N/A | N/A | 15 | -0.596 |
| KD07 | 20 | N/A | N/A | N/A | 15 | N/A |
| KD08 | 17 | -2.160 | N/A | N/A | 17 | -0.99 |
| KD09 | 17 | N/A | N/A | N/A | 17 | N/A |
| KD10 | / | / | / | / | / | / |
| KD11 | 21 | 0.260 | N/A | N/A | 21 | 1.140 |
| KD12 | 22 | 0.256 | N/A | N/A | 22 | 1.184 |
| KD13 | 22 | -0.413 | N/A | N/A | 21 | 0.360 |
| KD14 | 20 | N/A | N/A | N/A | 15 | N/A |
| KD15 | 22 | -1.077 | N/A | N/A | 22 | 0.192 |
| KD16 | 24 | 0.850 | N/A | N/A | 34 | 4.780 |
| KD17 | 20 | -0.634 | N/A | N/A | 16 | -0.841 |
| KD18 | 22 | 0.144 | N/A | N/A | 23 | 1.396 |
| KD19 | 23 | 0.200 | N/A | N/A | 20 | 0.200 |
| KD20 | 24 | 0.773 | N/A | N/A | 13 | -1.607 |
| KD21 | 23 | 0.494 | N/A | N/A | 21 | 0.826 |
| KD22 | 27 | 0.843 | N/A | N/A | 16 | -2.552 |
| KD23 | 21 | -1.201 | N/A | N/A | 17 | -1.190 |

N/A: Artery not visualized. /: Data loss.


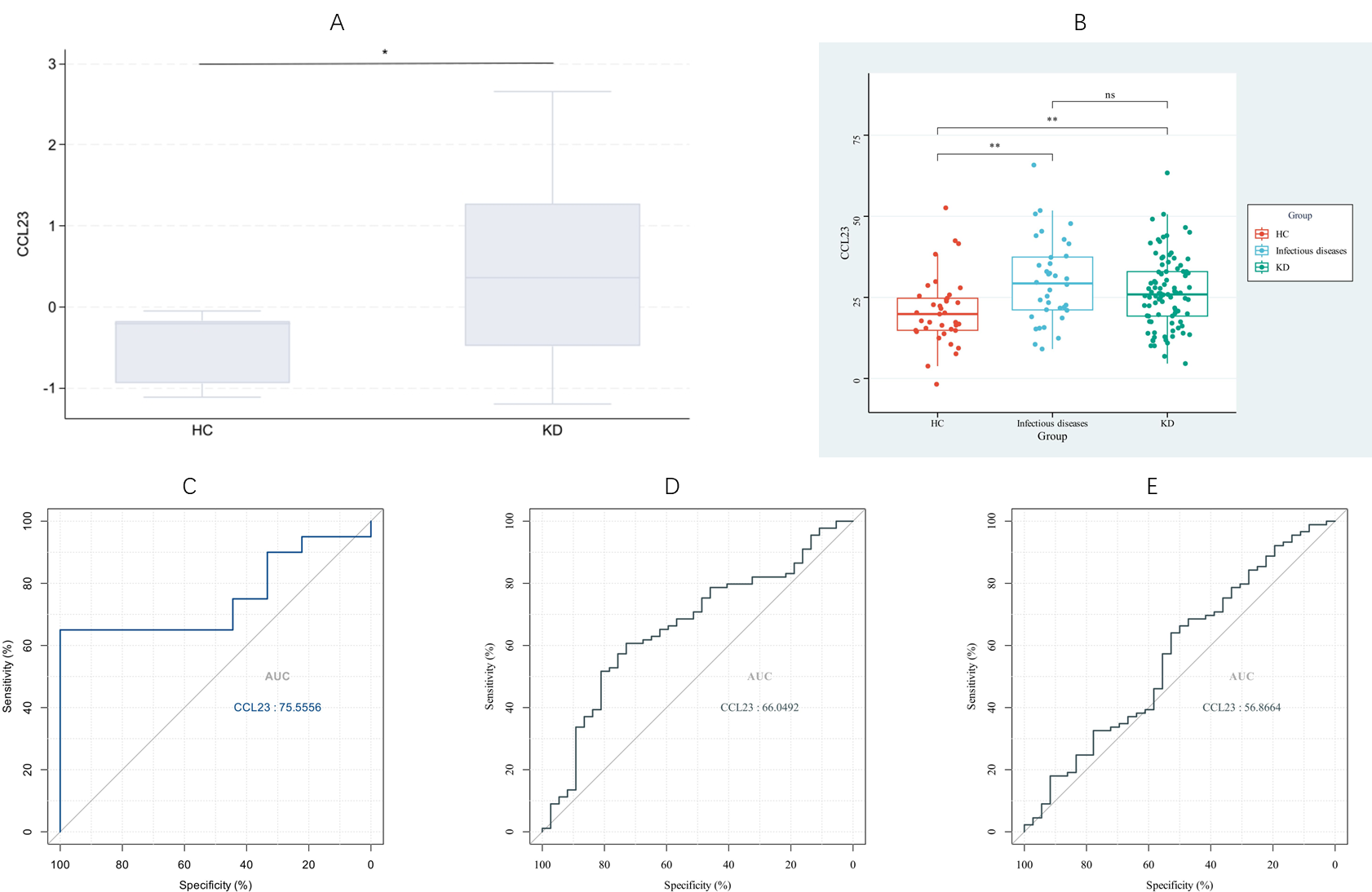


**Figure S1.** External validation results based on GEO datasets GSE18606 and GSE68004. HC, healthy control; KD, Kawasaki disease; AUC, area under the curve; CCL23, CC chemokine ligand 23. A: comparison between the two groups in the GSE18606 dataset; B: Comparison between the three groups in the GSE68004 dataset; C: ROC curve for distinguishing KD and HC in the GSE18606 dataset; D: ROC curve for distinguishing KD and HC in the GSE68004 dataset; E: ROC curve for distinguishing KD and infectious diseases in the GSE68004 dataset. Infectious diseases include adenovirus infection and Group A streptococcal disease.
